# Molecular subtypes and tumor microenvironment infiltration signatures based on cuproptosis-related genes in colon cancer

**DOI:** 10.3389/fonc.2023.999193

**Published:** 2023-05-18

**Authors:** Hongwei Luo, Que Zhang, Xiangchu Liu, Yue Luo, Xing Jiang, Chao Wang, Bin Chen, Qiming He, Yingchun Zhang, Ou Shu, Penggao Dai, Chengcheng He

**Affiliations:** ^1^ People’s Hospital of Mianzhu, Deyang, Sichuan, China; ^2^ People’s Hospital of Zhongjiang, Deyang, Sichuan, China; ^3^ Fujian Medical University, Fuzhou, Fujian, China

**Keywords:** colon cancer, cuproptosis, gene signature, molecular subtyping, immunity therapy, tumor microenvironment

## Abstract

**Background:**

Colon cancer is one of the common cancers, and its prognosis remains to be improved. The role of cuproptosis as a newly discovered form of cell death in the development of colon cancer has not been determined.

**Methods:**

Based on 983 colon cancer samples in the TCGA database and the GEO database, we performed a comprehensive genomic analysis to explore the molecular subtypes mediated by cuproptosis-related genes. Single-sample gene set enrichment analysis (ssGSEA) was utilized to quantify the relative abundance of each cell infiltrate in the TME. A risk score was established using least absolute shrinkage and selection operator regression (LASSO), and its predictive ability for colon cancer patients was verified to explore its guiding value for treatment.

**Results:**

We identified two distinct cuproptosis-related molecular subtypes in colon cancer. These two distinct molecular subtypes can predict clinicopathological features, prognosis, TME activity, and immune-infiltrating cells. A risk model was developed and its predictive ability was verified. Compared with patients in the high-risk score group, patients in the low-risk score group were characterized by lower tumor microenvironment score, higher stem cell activity, lower tumor mutational burden, lower microsatellite instability, higher sensitivity to chemotherapeutics, and better immunotherapy efficacy.

**Conclusion:**

This study contributes to understanding the molecular characteristics of cuproptosis-related subtypes. We demonstrate a critical role for cuproptosis genes in colon cancer s in the TME. Our study contributes to the development of individualized treatment regimens for colon cancer.

## 1 Introduction

Colon cancer is the third most common type in the world and the second leading cause of cancer-related death (1). Among all pathological subtypes, more than 90% of colorectal cancers are adenocarcinomas derived from colorectal mucosal epithelial cells. Cancer classification, prognosis prediction and treatment decisions by tumor, lymph node, metastasis (TNM) staging system and degree of histological differentiation have been frequently used. Because of the high degree of heterogeneity found in colon cancer, prognosis may vary significantly between patients despite similar clinical features. At present, the curative effect of surgical resection of the primary tumor combined with adjuvant chemotherapy for colon cancer patients through the TNM staging system is not satisfactory (2, 3). In recent years, with the development of cancer genomics, the classification of colon cancer has shifted from traditional histological subtypes to molecular subtypes. Guinney et al. divided colorectal cancer into four molecular subtypes including microsatellite unstable immune subtype, typical subtype, metabolic subtype and mesenchymal subtype through gene sequencing, and clarified the relationship between different subtypes and chemoresistance from a molecular perspective (4). There are also studies that type colon cancer by genetics. Zhu et al. divided colon cancer into three subtypes by autophagy-related genes, and demonstrated differences in prognosis and immunotherapy of different subtypes (5). Although these studies have deepened the understanding of colon cancer molecular subtypes, prediction of colon cancer patient prognosis based on these molecular subtypes remains less than satisfactory. Therefore, in order to more accurately stratify patients, it is necessary to develop new molecular subtypes. As a newly discovered way of cell death, many studies have shown that cuproptosis plays an important role in the regulation of cell death, which provides a new direction for the clinical treatment of colon cancer.

Cuproptosis is the direct binding of copper ions to fatty acylated components of the tricarboxylic acid cycle pathway, resulting in abnormal aggregation of fatty acylated proteins and loss of iron-sulfur cluster proteins, leading to proteotoxic stress responses and ultimately cell death (6). Copper ions and mitochondrial respiration in colon cancer have been found to be associated with various biological processes such as proliferation, drug resistance, and malignant transformation. Fat et al. found that elevated copper levels can induce oxidative stress in colon cancer cells and lead to cell apoptosis (7). Reprogramming of glucose metabolism is characteristic of cancer cells. Reduced mitochondrial respiration and enhanced glycolysis typically promote metastasis and inhibit apoptosis (8). In colon cancer, attenuated glycolysis and enhanced mitochondrial respiration inhibit cell growth (9). Therefore, we believe that the changes of intracellular copper ions in colon cancer and the cuproptosis pathways are of great significance for studying the prognosis of colon cancer patients and developing novel therapeutic targets.

In this study, we revealed the overall changes of cuproptosis-related genes (CRGs) at the transcriptional and genetic levels by downloading the transcriptional census data of colon cancer patients from TCGA database and GEO database, and then assessed the expression profiles of CRGs. First, patients were separated into two separate subtypes based on CRGs. Patients were also classified into two genotypes based on the DEG between the two subtypes. We further developed a scoring system to predict overall survival (OS) and describe the immune status, drug susceptibility, and immunotherapy effect of colon cancer.

## 2 Materials and methods

### 2.1 Data source

The gene expression profiling data of 437 COAD samples and corresponding clinical information were downloaded from TCGA database (https://portal.gdc.cancer.gov/). Gene expression profiling data included 39 normal samples and 398 tumor samples. Clinical data included age, gender, histological grade, survival time and pathological stage (**Table 1**). The GSE39582 dataset was downloaded from the Gene Expression Omnibus (GEO), which includes gene expression information and clinical information for 585 patients (GEO, https://www.ncbi.nlm.nih.gov/geo/). FPKM values for TCGA colon cancer were converted to transcripts per kilobase million (TPM), as this was considered to be the same transcript as the microarray in the GEO dataset (10, 11). The two datasets were combined for subsequent analysis, and batch effects were eliminated by applying the “Combat” algorithm.

**Table 1:** The clinical characteristics of colon cancer patients.

Characteristics	Total sample	
	TCGA cohort(N=385)	GSE39582(N=556)
Age		
<=60	112	157
>60	273	399
Gender		
male	205	307
female	180	249
AJCC Stage		
I	66	32
II	151	258
III	103	203
IV	54	59
unknown	11	4
T stage		
T1	9	11
T2	68	44
T3	263	360
T4	44	117
T0/Tis	1	4
unknown	0	20
N		
N0	231	295
N1	88	131
N2	66	98
N3	0	6
unknown	0	26
M stage		
M0	286	474
M1	54	30
unknown	45	22
Fustat		
alive	306	369
dead	79	187
Chemotherapy adjuvant		
Y	NA	240
N	NA	316
Chemotherapy adjuvant type		
5FU	NA	82
FOLFIRI	NA	12
FOLFOX	NA	77
unknown	NA	69

AJCC, American Joint Committee on Cancer.

### 2.2 Gene mutation analysis

Somatic mutation data were downloaded from the TCGA database and visualized by the “maftools” package in R language. The waterfall plot shows mutation information for each gene. In the upper right corner of the waterfall chart, the different mutation types are marked with different colors.

### 2.3 Identification of differentially expressed cuproptosis-related genes

Gene difference analysis was performed using the “limma” package on the R language. False discovery rate (FDR) <0.05 and |log2 fold change (log2FC)|≥0.585 were set as cutoff values for screening DEGs. The intersection of DEGs and cuproptosis-related genes was considered as a group of significantly differentially expressed CRGs.

### 2.4 Consensus clustering analysis of CRGs

Nineteen genes associated with cuproptosis were identified from previous studies. Consistent unsupervised clustering analysis was performed using the R software package “ConsensusClusterPlus” to classify patients into distinct molecular subtypes based on CRG expression. And 1000 cycles were carried out to ensure the stability of the classification (12). This clustering is performed based on the following criteria: First, the cumulative distribution function (CDF) curve grows gradually and smoothly. Second, no group has a small sample size. Then principal component analysis (PCA) based on hub genes was performed using the “ggplot2” package, and two-dimensional PCA plots were drawn. Finally, after clustering, intra-group correlations increase and inter-group correlations decrease. To investigate the differences of CRGs in biological processes. Gene Set Variation Analysis (GSVA) was performed using the hallmark gene set (c2.cp.kegg.v7.2) in the MSigDB database.

### 2.5 Construction and validation of a prognostic model based on cuproptosis-related genes

The prognostic model was constructed based on the target dataset and randomly divided into a training cohort and a validation cohort. The initial screen of CRG-related genes associated with prognosis was further narrowed by least absolute shrinkage and selection operator (LASSO) analysis using the glmnet R package. The constructed prognostic model can be simply expressed as: risk score=∑(β1*Exp1+β2* Exp2 +β3* Exp3+⋯+βn* Expn) (β: coefficients, Exp: gene expression level), where X represents the expression level of each CRGs, and Coef represents the coefficient of relative prognostic CRGs in the multivariate Cox regression model. From the prognostic model, a prognostic risk score can be calculated for each colon cancer patient, and moderation is defined as the boundary between the high- and low-risk groups. Patients with risk scores above the median were classified as high risk. Furthermore, the predictive performance of the constructed predictive model is validated by a validation cohort.

### 2.6 Tumor microenvironment, stem cell characteristics and drug susceptibility analysis.

Infiltration levels of immune cells and stromal cells in different tumor tissues were analyzed by immune score and stromal score. Spearman’s correlation was used to test the correlation between risk scores and these scores. Associations between risk scores and immune infiltrating subtypes were examined by 2-way ANOVA analysis. Tumor stem cell signatures extracted from the transcriptome and epigenetics of TCGA tumor samples were used to measure stem cell-like characteristics of tumors. The Spearman correlation test was used to analyze the correlation between tumor stemness and risk score. In order to explore the difference in the treatment effect of chemotherapeutic drugs in the two groups of patients, we calculated the half-inhibitory concentration (IC50) value of the commonly used chemotherapeutic drugs using the “pRRophetic” software package.

### 2.7 Immunotherapy cohort collection

Imvigor 210 (http://research-pub.gene.com/IMvigor 210CoreBiology) is a cohort of urothelial cancer patients treated with PD-L1 (12). It has relatively complete survival information, follow-up information and immunotherapy effective information. Excluding samples with incomplete clinical data, 298 samples were finally obtained for follow-up analysis. Raw count data were normalized using the DEseq2 R package. In addition, differential analysis of immune checkpoints in high and low risk groups were performed.

### 2.8 Statistical analysis

Statistical analysis Wilcoxon test was used to compare DEGs of tumor tissue and normal samples. Survival and survminer packages in R language were used for survival analysis. The chi-square test was used to compare different proportions. Comparison of ssGSEA scores of immune cells or immune pathways between high-risk and low-risk groups was performed by the Mann-Whitney test. Correlations of prognostic model risk scores or prognostic gene expression levels with stem cell scores, stromal scores, immune scores, and drug sensitivity were detected by Spearman or Pearson correlation analysis. Plots were created using the R software (version 4.0) of the packages graph, ggplot2, pheatmap, ggpubr and corrplot. In all statistical results, P<0.05 indicates statistical significance.

## 3 Result

### 3.1 Genetic and transcriptional alterations of CRGs in colon cancer

**Figure 1:**
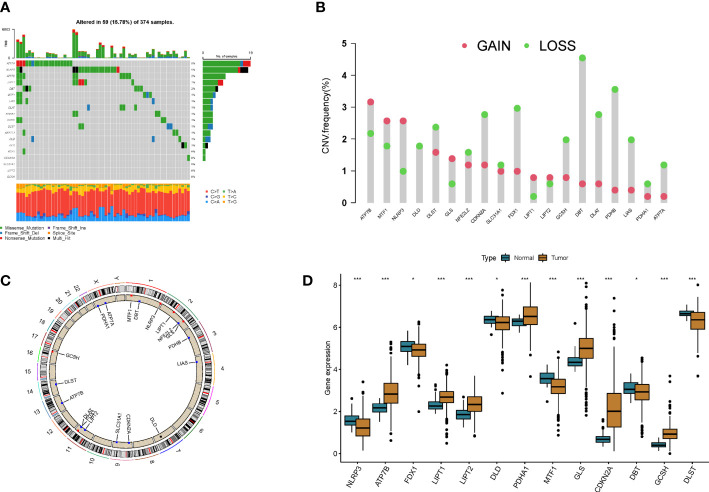
Genetic and transcriptional alterations in cuproptosis-related genes in colon cancer. **(A)** Mutation frequencies of 19 cuproptosis-related genes in colon cancer patients. **(B)** Frequency of CNV changes in cuproptosis-related genes. **(C)** Location of CNV alterations in cuproptosis-related genes on 23 chromosomes. **(D)** Expression distribution of 19 cuproptosis-related genes in normal and colon cancer tissues *p<0.05, ***p<0.001.

A total of 19 CRGs were included in this study. By summarizing the somatic mutation frequency of 19 CRGs, we found that ATPA7 and NLRP3 had the highest mutation frequency in colon cancer (5%; **Figure 1A**). Next, we investigated somatic copy number alterations in these CRGs. We further compared mRNA expression levels and found copy number alterations in all 19 CRGs. Among them, ATP7B, MTF1, and NLRP3 had extensive copy number variation (CNV) increases, while the remaining CRGs showed CNV decreases (**Figure 1B**). **Figure 1C** shows the location of CNV alterations in GRGs on their respective chromosomes. Expression levels of most CRGs were found to be negatively correlated with changes in CNVs. This suggests that CNV may be associated with regulating the mRNA expression of CRGs. By analyzing the expression of CRGs between tumor samples and control samples, we found significant differences in the expression levels of CRGs, suggesting a potential role of CRGs in colon carcinogenesis (**Figure 1D**).

### 3.2 Identification of cuproptosis subtypes in colon cancer

**Figure 2:**
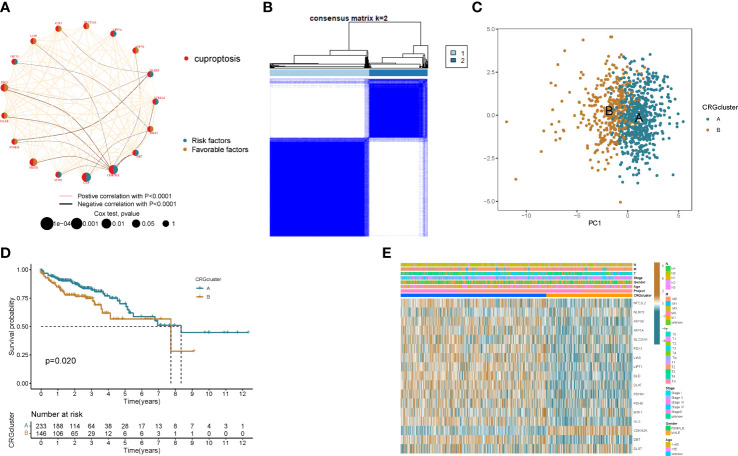
Identification of cuproptosis subtypes in colon cancer. **(A)** Interactions between CRGs in colon cancer. Lines connecting PRGs indicate their interactions. **(B)** Consensus matrix heatmap defining two clusters (k = 2) and their correlation area. **(C)** Principal component analysis shows that the two subtypes are distinct. **(D)** Kaplan–Meier curve of OS between the two cuproptosis subtypes. **(E)** Comparison of the distribution of patients with different clinicopathological features between the two cuproptosis subtypes.

To fully understand the expression patterns of CRGs in tumorigenesis, we included 967 patients in TCGA and GEO into our study for further analysis. Results of Kaplan–Meier analysis showed the prognostic value of CRGs in colon cancer patients (**Figures S1**, **S2**). Next, the 17 CRGs were constructed into a network graph, enabling a comprehensive analysis of gene interactions and interconnections and their impact on the prognosis of colon cancer patients (**Figure 2A**). To further explore the expression characteristics of CRGs in colon cancer, we used a consensus clustering algorithm to classify colon cancer patients based on the expression profiles of 17 prognosis-related CRGs (**Figure S3**). Our results indicated that k = 2 appeared to be the best choice for dividing the entire cohort into A subtype (n = 608) and B subtype (n = 359) (**Figure 2B**). PCA analysis revealed significant differences in the transcriptional profiles of CRGs between the two isoforms (**Figure 2C**). Kaplan–Meier curves showed that patients with subtype B had a longer recurrence free survival (RFS) than patients with subtype A (p = 0.02; **Figure 2D**). Furthermore, comparing the clinicopathological features of different subtypes of colon cancer revealed significant differences in CRG expression and clinicopathological features (**Figure 2E**). This further demonstrates that CRGs can be used to distinguish patients with different clinical features.

### 3.3 Characteristics of TME cell infiltration and biological function in the cuproptosis subtypes

**Figure 3:**
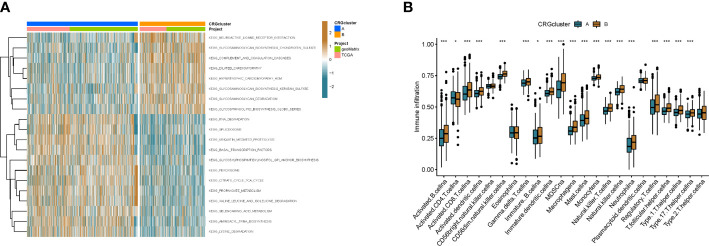
The immune landscape of two colon cancer subtypes. **(A)** Differences in pathway activity between the two different subtypes were reflected using GSVA scores. **(B)** Abundance of 23 infiltrating immune cell types in the two colon cance subtypes. *p<0.05, ***p<0.001.

To better understand the difference in survival between the 2 clusters. The 2 subtypes were first subjected to GSVA enrichment analysis to examine their functional and biological differences (**Figure 3A**). Through the comparative analysis of the enrichment of cluster A and cluster B, the results show that cluster A is mainly enriched in the metabolic pathways of nucleotides and amino acids, such as RNA degradation, lysine degradation, citrate cycle tca cycle, selenoamino acid metabolism and valine leucine and isoleucine degradation; cluster B is mainly related to the metabolic pathways of sugars, such as glycosaminoglycan degradation, glycosphingolipid biosynthesis globo series and glycosaminoglycan biosynthesis chondroitin sulfate. This suggests that metabolism may be more active in cluster A and gluconeogenesis is involved, which is consistent with cellular cuproptosis features. Next, to investigate the role of CRGs in the TME of colon cancer patients, ssGSEA analysis was used to assess the enrichment fractions of 23 immune cells in the 2 subtypes. We observed significant differences in the infiltration of most immune cells between the two subtypes (**Figure 3B**). The infiltration level of Activated CD4 T cells in cluster A was significantly higher than that in cluster B, while Activated B cell, Activated CD8 T cell, Activated dendritic cell, CD56dim tural killer cell, Gamma delta T cell, Immature B cell, Immature dendritic cell, Infiltration of MDSC, Macrophage, Mast cell, Monocyte, tural killer T cell, tural killer cell, Neutrophil, Regulatory T cell, T follicular helper cell, Type 1 T helper cell, Type 17 T helper cell, Type.2 T helper cell significantly reduced.

### 3.4 Identification of gene subtypes based on DEGs

**Figure 4:**
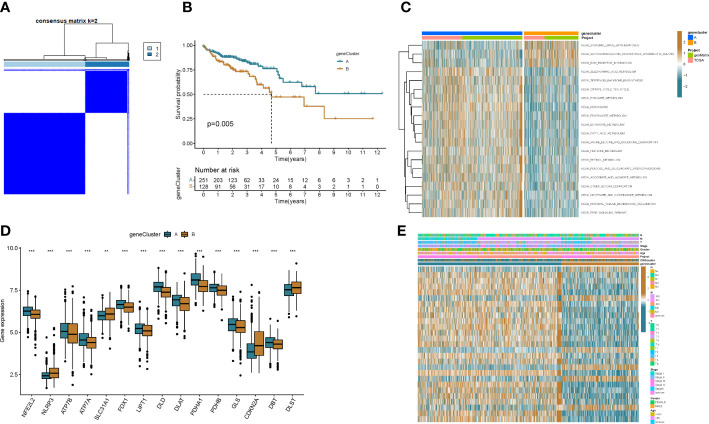
Genotyping based on DEGs. **(A)** Consensus matrix heatmap defining two clusters (k = 2) and their correlation area. **(B)** Kaplan–Meier curve of OS between two gene subtypes. **(C)** Differences in pathway activity between the two different subtypes were reflected using GSVA scores. **(D)** Differences in expression of CRGs between the two genotypes. **(E)** Association between clinicopathological features and the two genotypes. **p<0.01, ***p<0.001.

To explore the underlying biological behavior of each ptosis pattern, we identified 160 DEGs associated with cuproptosis subtypes using the R package “limma”. We then performed univariate Cx regression analysis to determine the prognostic value of 160 subtype-related genes and screened out 27 genes (p < 0.05) associated with RFS time for subsequent analysis (**Table S1**). To further validate this regulatory mechanism, patients were divided into 2 genomic subtypes based on prognostic genes using a consensus clustering algorithm (**Figure 4A** and **Figure S3**). Kaplan-Meier curves showed that patients with genotype B had worse RFS than patients with gene cluster A (p=0.005; (**Figure 4B**). GSVA enrichment analysis showed that the gene cluster A was mainly enriched in gluconeogenesis and metabolic activity-related pathways, including citrate cycle tca cycle, pyruvate metabolism, butanoate metabolism, fatty acid metabolism, valine leucine and isoleucine degradation, etc (**Figure 4C**). These results indicate that patients with metabolically active genomic subtypes have a better prognosis. And by differential analysis we found that most of the CRGs were highly expressed in the patients of the gene cluster A (**Figure 4D**). This suggests that metabolically active tumor cells can trigger cuproptosis in cells, thereby improving patient outcomes. In addition, the gene cluster B pattern correlated with advanced TNM stage (**Figure 4E**).

### 3.5 Construction of a prognostic model

**Figure 5:**
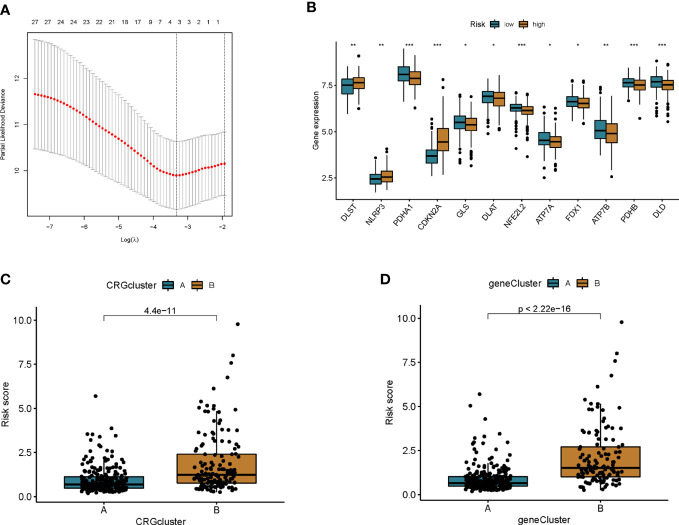
Construction of prognostic features of colon cancer patients. **(A)** Cross-validation for optimal penalty parameter selection in the LASSO model. **(B)** Differences in CRG expression between high and low risk groups. **(C)** Risk score differences between cuproptosis subtypes. **(D)** Differences in risk scores between genotypes. *p<0.05, **p<0.01, ***p<0.001.

**Figure 6:**
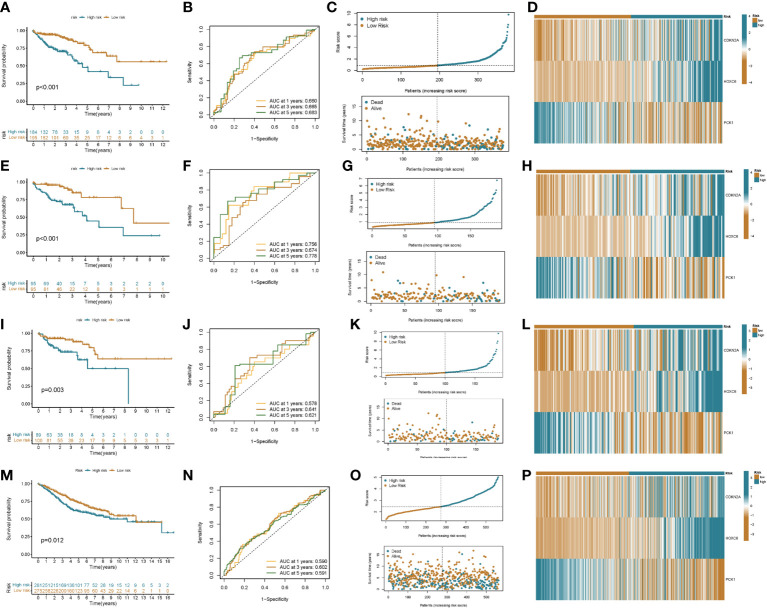
Prognostic value of risk score in colon cancer patients. Kaplan–Meier analysis of RFS between high and low risk groups (**A**: all patients; **E**: training group; **I**: validation group; **M**: GEO cohort). ROC curves for sensitivity and specificity for predicting 1-, 3-, and 5-year survival between high and low risk groups (**B**: all patients; **F**: training group; **J**: validation group; **N**: GEO cohort). Ordinal dot and scatter plots of risk score distribution and patient survival status (**C**: all patients; **G**: training group; **K**: validation group; **O**: GEO cohort). Heat map of risk-related genes between high and low risk groups (**D**: all patients; **H**: training group; **L**: validation group; **P**: GEO cohort).

A prognostic model was established based on subtype-related DEGs. First, we randomly divided patients into training and testing groups in a 1:1 ratio using the “caret package” in R. LASSO and multivariate Cox analyses were performed on the 27 prognostic DEGs associated with ptosis subtypes to further select the best prognostic markers. Finally, a 3-gene prognosis prediction model was obtained (**Figure 5A**). The risk score was calculated as follows: risk scores = 0.4193*CDKN2A expression level 0.293*HOXC6 expression level+-0.162*PCK1 expression level. Patients were subdivided into high-risk and low-risk groups according to the median cutoff value. The results of differential analysis of CRGs showed that PDHA1, GLS, DLAT, NFE2L2, ATP7A, FDX1, ATP7B, PDHB, and DLD were highly expressed in the low-risk group, while DLST, NLRP3, and CDKN2A were low-expressed (**Figure 5B**). Subsequently, we analyzed the risk relationship between score and cuproptosis typing and genotyping. The results showed that subtype A, which had a better prognosis, had lower risk scores in cuproptosis subtyping (**Figure 5C**), and similar results were obtained in genotyping (**Figure 5D**). Through independent prognostic analysis, we identified the correlation between clinical characteristics, risk score and prognosis (**Table 2)**. Next, we performed a survival analysis on samples from the total sample, training set, and validation set, and the results showed that among the four groups, the prognosis of the low-risk group was better than that of the high-risk group (p<0.05; **Figures 6A, E, K, O**). Furthermore, using the time-dependent ROC curve to analyze the survival prediction of the prognostic model, the area under the curve (AUC) in the total sample reached 0.66 at 1 year, 0.665 at 3 years, and 0.683 at 5 years (**Figure 6B**). The area under the curve (AUC) in the training group reached 0.756 at 1 year, 0.674 at 3 years, and 0.778 at 5 years (**Figure 6H**). The area under the curve (AUC) in the validation group reached 0.578 at 1 year and 0.641 at 3 years, reaching 0.621 at 5 years (**Figure 6L**). The area under the curve (AUC) in the GEO cohort reached 0.590 at 1 year and 0.602 at 3 years, reaching 0.591 at 5 years (**Figure 6P**). The risk curve shows that as the risk score increases, the number of deaths of patients in each group also increases (**Figures 6C, I, M, Q**). High expression in the high-risk group, and low expression of protective genes in the high-risk group (**Figures 6D, J, N, R**).

**Table 2:** Independent prognostic analysis of clinical characteristics and risk scores.

clinical characteristics	HR	HR.95L	HR.95H	pvalue
Age	1.031684	1.008779	1.05511	0.00646991
Gender	1.00004	0.615046	1.626023	0.999871903
Stage	2.456277	1.849692	3.261785	5.30E-10
riskScore	1.253685	1.106866	1.419977	0.000374158

### 3.6 TME and cancer stem cells in high-risk and low-risk groups

**Figure 7:**
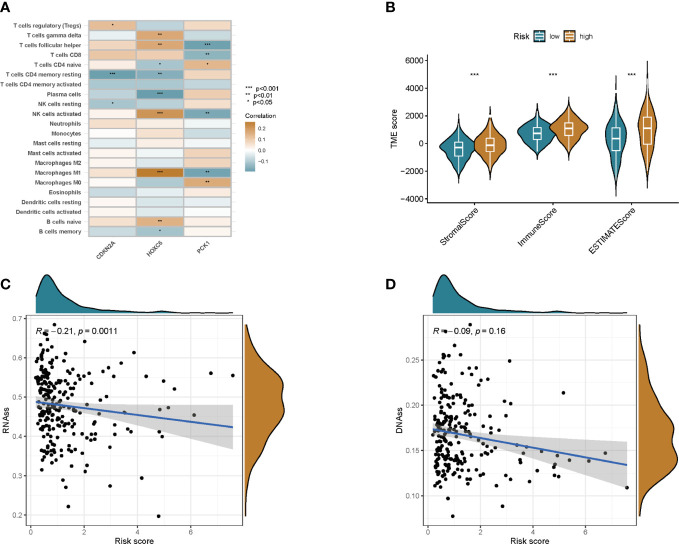
TME and stem cell activity were assessed between the two groups. **(A)** Correlation between immune cell abundance and three genes in this model. **(B)** Correlations between risk scores and immune and stromal scores. **(C)** Correlation of risk scores with RNAss. **(D)** Correlation of risk score with DNAss. *p<0.05, **p<0.01, ***p<0.001.

We used the CIBERSORT algorithm to assess the association between risk score and immune cell abundance. The results showed that the risk score was positively correlated with T cells follicular helper, T cells CD8, NK cells activated, and Macrophages M1, and negatively correlated with T cells CD4 memory resting, Plasma cells, NK cells resting, and B cells memory (**Figures S5A–H**). We also evaluated the relationship between 3 genes in this model and immune cell abundance. We observed that HOXC6 correlated most strongly with immune cell abundance (**Figure 7A**). Furthermore, the StromalScore, ImmuneScore, and ESTIMATEScore were lower in the low-risk group than in the high-risk group (**Figure 7B**).

Cancer stem cells can be measured by RNA stem cell score (RNAss) based on mRNA expression and DNA stem cell score (DNAss) based on DNA methylation patterns (13). The correlation between prognostic gene expression and cancer stem cells was analyzed, and the results showed that the prognostic model was negatively correlated with RNAss, but not with DNAss (**Figures 7C, D**).

### 3.7 Drug susceptibility and immunotherapy analysis

**Figure 8:**
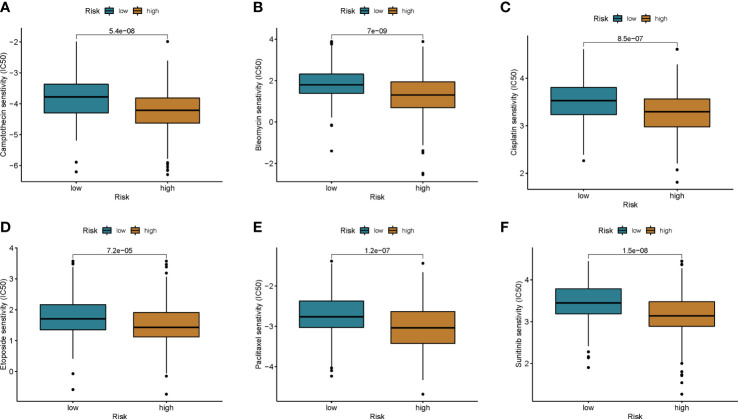
Chemotherapy drug sensitivity was assessed between the two groups. **(A)** Camptothecin, **(B)** Bleomycin, **(C)** Cisplatin, **(D)** Etoposide, **(E)** Paclitaxel, and **(F)** Sunitinib.

**Figure 9:**
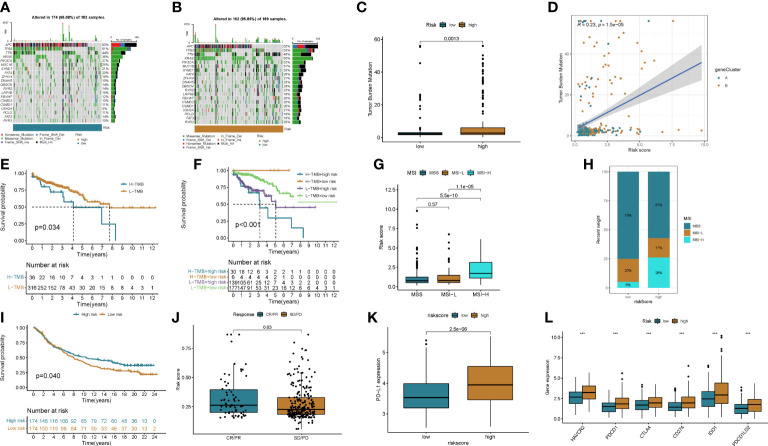
The effect of immunotherapy between the two groups was assessed. **(A, B)** Waterfall plots of somatic mutation signatures of patients in the high- and low-risk groups. Each column represents a patient. The upper bar graph shows TMB, and the numbers on the right represent the mutation frequency of each gene. The right bar graph shows the proportion of each variant type. **(C, D)** Differences in TMB in high- and low-risk groups and correlation of risk scores with TMB. **(E, F)** Kaplan–Meier analysis of RFS between TMB and high- and low-risk groups. **(G, H)** Relationship between risk score and MSI. **(I)** Kaplan-Meier plot of overall survival in the cuproptosis score group of patients in the IMvigor210 cohort. **(J)** Correlation of risk score with response to anti-PD-L1 immunotherapy. PR, partial response; PD, progressive disease; SD, stable disease; CR, complete remission. **(K)** Differential expression of PD-L1 in high and low risk groups. **(L)** Expression of immune checkpoints in the high and low-risk groups. ***p<0.001.

To explore potential treatment options for patients in different risk groups. We analyzed the sensitivity of colon cancer patients to commonly used chemotherapeutic agents and showed that the sensitivity to Camptothecin, Bleomycin, Cisplatin, Etoposide, Paclitaxel, and Sunitinibwas significantly higher in patients in the low-risk group than in the higher-risk group (**Figures 8A–F**). Some studies have shown that, colon cancer patients with high tumor mutational burden (TMB) values may have poor prognosis (14). However, the higher the TMB, the better the tumor remission and clinical benefit of immunotherapy (14). We therefore analyzed the TMB of patients in the high and low risk groups in the TCGA cohort. The top ten mutated genes in the high-risk and low-risk groups were APC, TP53, TTN, KRAS, PIK3CA, MUC16, SYNE1, FAT4, ZFHX4, and DNAH5 (**Figures 9A, B**). Among patients in the high and low risk groups, TMB was different and positively correlated with risk scores (**Figures 9C, D**). The results of survival analysis indicated that patients with high TMB had poor prognosis, which was consistent with previous studies (**Figures 9E, F**). There is increasing evidence that patients with high microsatellite instability (MSI-H) are more sensitive to immunotherapy and may benefit from immunotherapy drugs (15).

We found that MSI-H was positively correlated with risk score, and MSI-H in high-risk group patients was significantly higher than that in low-risk group patients (**Figures 9G, H**).

The results of differential analysis showed that PD-1 expression in patients in the high-risk group was higher in the lower-risk group (**Figure 9I**). We next downloaded a dataset of patients receiving immunotherapy from the Imvigor 210 database. The prognosis of patients in the high- and low-risk groups in this dataset was analyzed, and the results showed that the low-risk group had a better prognosis than the high-risk group (**Figure 9J**). By analyzing patients’ response to immunotherapy, we found that the efficacy of immunotherapy was positively correlated with the risk score (**Figure 9K**). We then analyzed the differences in immune checkpoint expression between patients in the high and low risk groups, and the results showed that most immune checkpoints were highly expressed in patients in the high-risk group (**Figure 9L** and **Figure S6A**).

The above results suggested that chemotherapy may be a potential treatment modality for patients in the low-risk group. For patients in the high-risk group, immunotherapy may be more promising. And a risk score that can be used to predict the efficacy of immunotherapy.

## 4 Discussion

With the advancement of technology and the application of new treatment methods, the treatment strategy of colon cancer has made important progress, but the prognosis of some patients is still poor, and the average five-year survival rate of advanced patients is <30% (16). Therefore, it is necessary for clinical decision-making to accurately identify the molecular subtypes and their clinical characteristics of colon cancer, and to construct an effective prognostic prediction model to find potential therapeutic targets. Cuprotosis is a newly discovered form of cell death that has been shown to play an important role in the development of human disease (17). Although there is no research on copper drooping in the occurrence and development of colon cancer so far. However, numerous studies have demonstrated the important role of copper in colon cancer cells (7, 9). We investigated the integrated roles of these cuprotosis-related genes in colon cancer molecular typing microenvironment cell infiltration properties and immunotherapy.

In this study, we divided colon cancer patients into two subtypes based on CRG. The two subtypes exhibit markedly different clinical features, immune status, biological processes, and outcomes. In order to clarify the gene regulatory relationship between the two subtypes, we performed differential expression analysis based on the genes of the two subtypes. We then screened out the prognosis-related DEGs, and divided the patients into two genotypes by the prognosis-related DEGs. The results of the analysis of the two genotypes showed that the genotype of CRG can be used as an indicator for predicting prognosis and identifying clinical features at the gene level. Therefore, we combined LASSO regression and multivariate Cox regression analysis to screen for the most informative prognostic indicators that might constitute the final features. Ultimately, we derived a prognostic risk signature that showed good predictive power. Through the analysis of risk scores, we found that the expression levels of CRG in different scoring groups were significantly different. And the risk grouping is consistent with CRG subtype and genotype. We then analyzed the relationship between risk score and tumor microenvironment and tumor stem cell properties to clarify the correlation between risk score and tumor malignancy. Finally, mutations, immune checkpoints, MSI, drug susceptibility, and immunotherapy were analyzed in patients in the high and low risk groups. This helps to provide recommendations for the selection of treatment options for colon cancer patients in high and low risk groups.

Regardless of the fact that surgery combined with radiotherapy and chemotherapy has greatly improved the therapeutic effect of colon cancer patients, the prognosis varies greatly due to the heterogeneity of patients. Therefore, individualized treatment is of particular importance. The tumor microenvironment (TME) is deemed to be an an important factor affecting tumor cell proliferation, spread, drug resistance and immunotherapy (15, 18, 19). Typical structures of the TME include immune and inflammatory cells, endothelial cells, myofibroblasts, fibroblasts, adipocytes, and extracellular matrix (20). In this study, we performed a thorough analysis of the association between genes involved in risk scoring and TME immune activity. The results showed that the high-scoring group was associated with immune activation in the tumor microenvironment, and the low-scoring group was associated with immunosuppression in the tumor microenvironment. Macrophages may play a significant role in promoting tumor cells in the TME by inhibiting T cell-mediated anti-tumor immune responses (21, 22). At the primary site of the tumor, macrophages will directly enhance tumor cell growth by promoting angiogenesis, or indirectly induce dysfunction of immune cell interactions within the TME (23). Tumor-infiltrating B lymphocytes inhibit tumor progression by secreting immunoglobulins, promoting T cell responses, and directly killing cancer cells (24). In the present study, we observed higher macrophage infiltration and lower B cell infiltration in subtype B and high risk score groups, which may be associated with poor prognosis. CD8+ T cells play a major role in directly killing tumor cells by recognizing tumor antigens and are important effector cells for immunotherapy (25). Natural killer cells can select new tumor-killing cells. Activation of NK cells in colon cancer has a positive impact on improving patient outcomes (26). CD8+ T cells, T cells follicular helper, and NK cell infiltration were elevated in high-risk and B subtypes, suggesting that immunotherapy may be a potential treatment option for patients with poor prognosis.

Immunotherapy includes immune checkpoint inhibitors (ICIs), therapeutic antibodies and cell therapy. Currently, ICIs are utilized to treat colorectal cancer with satisfactory results (3, 27). Biomarkers such as programmed cell death ligand 1 (PD-L1), tumor mutational burden (TMB) and microsatellite instability high (MSI-H)/mismatch repair deficiency (dMMR) have been shown to be predictors of ICIs antitumor efficacy (28–31). In the present study, we observed higher expression levels of PD-L1 in patients in the high-risk group. By analyzing immune checkpoint molecules, we found that the expression levels of CD274, PDCD1, PDCD1LG2, CTLA4, HAVCR2, and IDO1 in the high-risk group were higher than those in the low-risk group. Microsatellite instability high (MSI-H)/mismatch repair deficiency (dMMR) has been shown to be a predictor of ICI anti-tumor efficacy, and we observed higher proportions of MSI-H and dMMR in the high-risk group than in the low-risk group. Then, the relationship between the cuproptosis score and the efficacy of immunotherapy was further analyzed in the imvigor210 cohort of metastatic urothelial carcinomas receiving anti-PD-L1 therapy. CR/PR rates were positively correlated with risk scores. This is direct evidence of better immunotherapy outcomes in patients with high risk scores. We then analyzed the sensitivity of patients with different risk groups to common chemotherapeutic agents for colon cancer. The results showed that the sensitivity of patients in the low-risk group to Camptothecin, Bleomycin, Cisplatin, Etoposide, Paclitaxel, and Sunitinib was higher than that of the patients in the high-risk group. Therefore, we believe that the cuproptosis score can be used to develop a personalized treatment plan for the patient. Patients in the low-score group chose chemotherapy, while immunotherapy was a more effective treatment option for patients in the high-score group.

The study still has limitations. All analyses were performed only on data from public databases, and all samples used in our study were obtained retrospectively. Therefore, follow-up prospective studies combined with *in vitro* and *in vivo* experiments are extremely important to improve the clinical significance of this study.

## 5 Conclusions

In conclusion, this study clarified the relationship between CRG and tumor immune matrix microenvironment, clinicopathological features and prognosis by comprehensive analysis of CRG expression profiles in colon cancer patients. We also determined the therapeutic role of CRG in chemotherapy and immunotherapy. These findings highlight the important clinical significance of CRG and provide new ideas for guiding personalized treatment strategies for patients with colon cancer.

## Data availability statement

The original contributions presented in the study are included in the article/**Supplementary Material**. Further inquiries can be directed to the corresponding authors.

## Author contributions

CH, HL, and PD conceived the project. HL, QZ, XL, YL, XJ, CW, BC and QH contributed to data acquisition, analysis and interpretation, and manuscript writing. OS and PD revised the manuscript. All authors contributed to the article and approved the submitted version.
