# Supplementary material for: Molecular subtypes and tumor microenvironment infiltration signatures based on cuproptosis-related genes in colon cancer
Source: Front Oncol. 2023 May 18;13:999193. doi: 10.3389/fonc.2023.999193 (PMC10234596; doi:10.3389/fonc.2023.999193)
Supplement: Supplementary file 1 [file DataSheet_1.pdf]

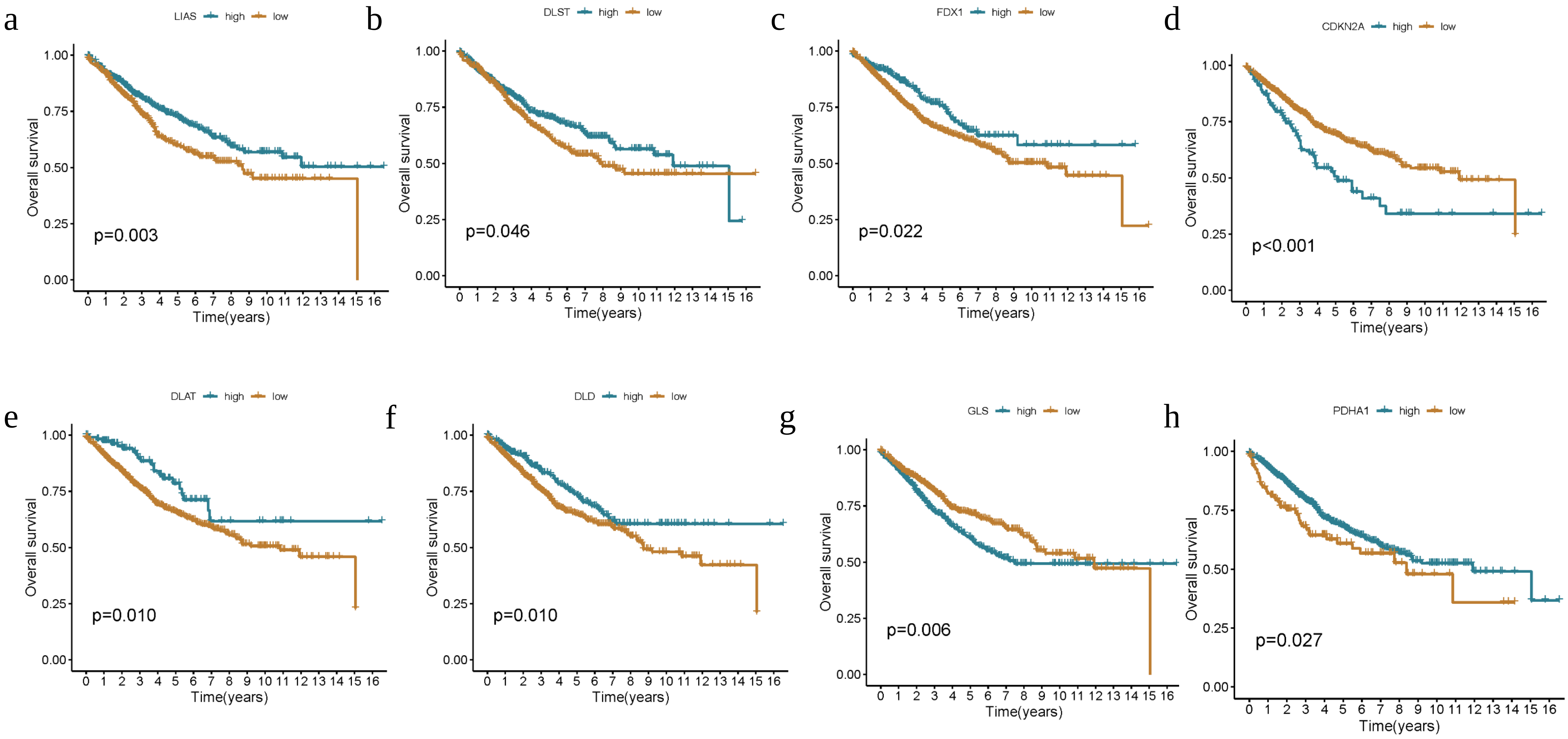

Figure S1 Relationship between CRGs and prognosis

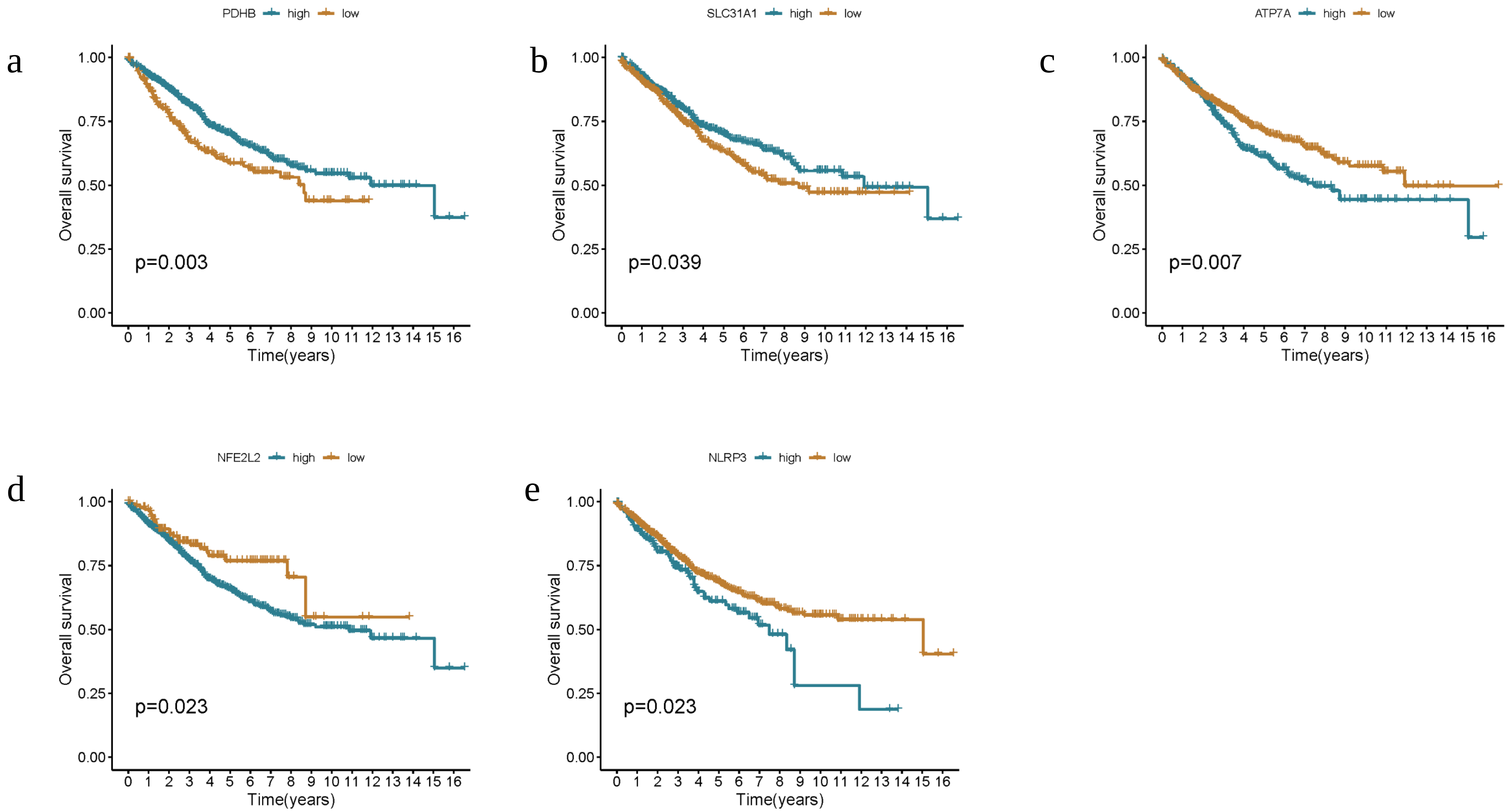

Figure S2 Relationship between CRGs and prognosis

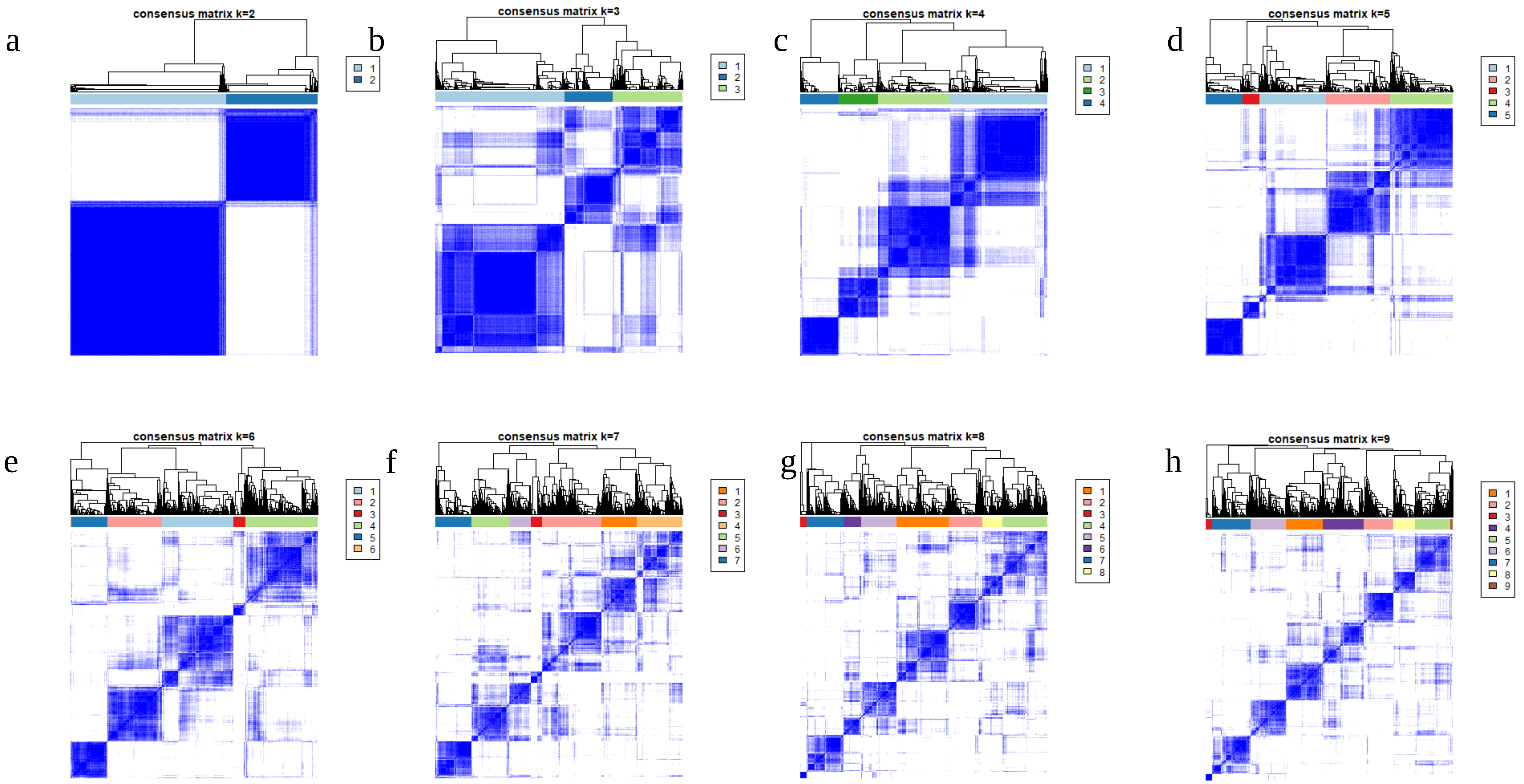

Figure S3 Cuproptosis subtypes' consensus matrix heatmap and their correlation area.

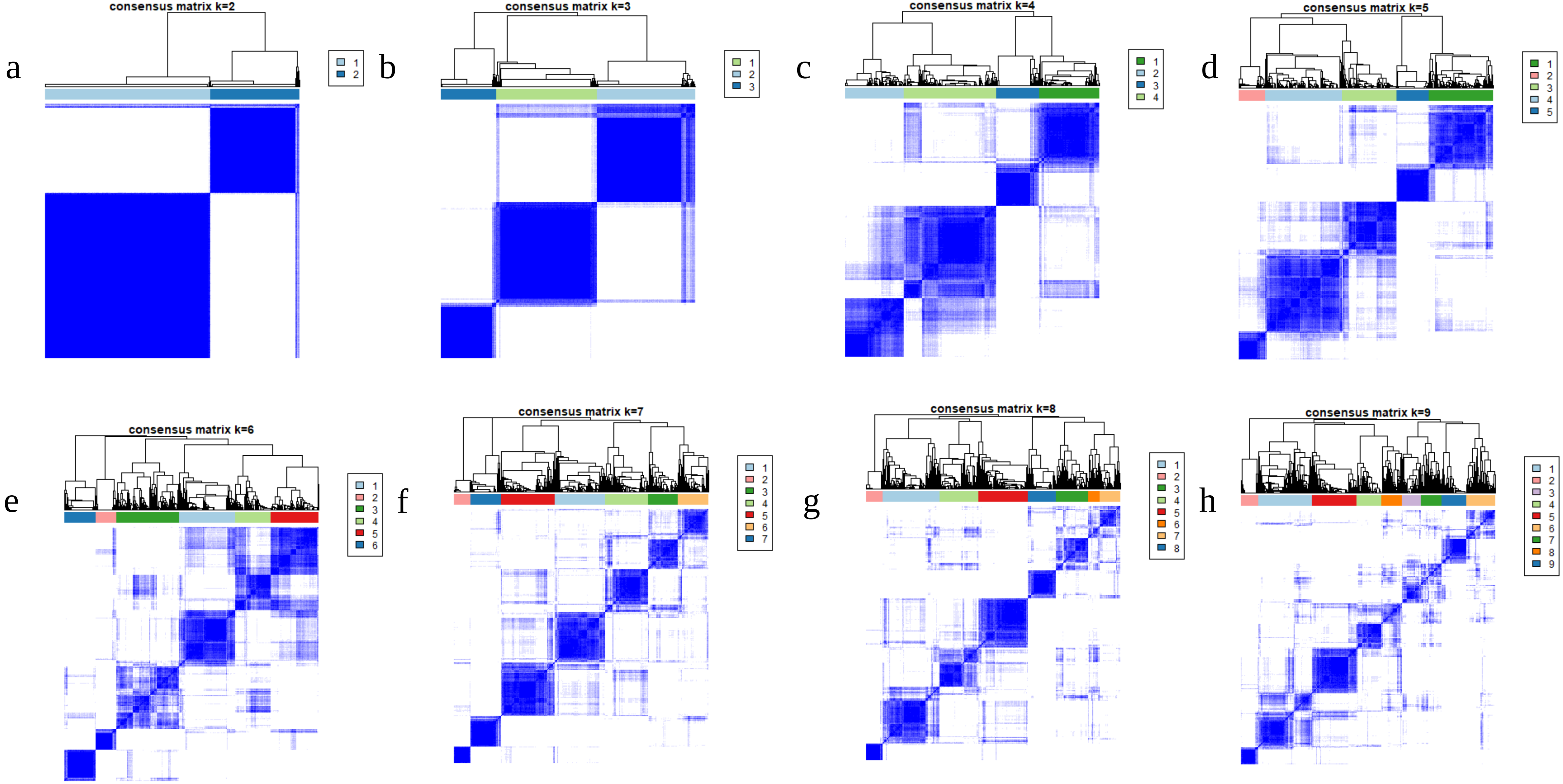

Figure S4 Gene subtypes' consensus matrix heatmap and their correlation area.

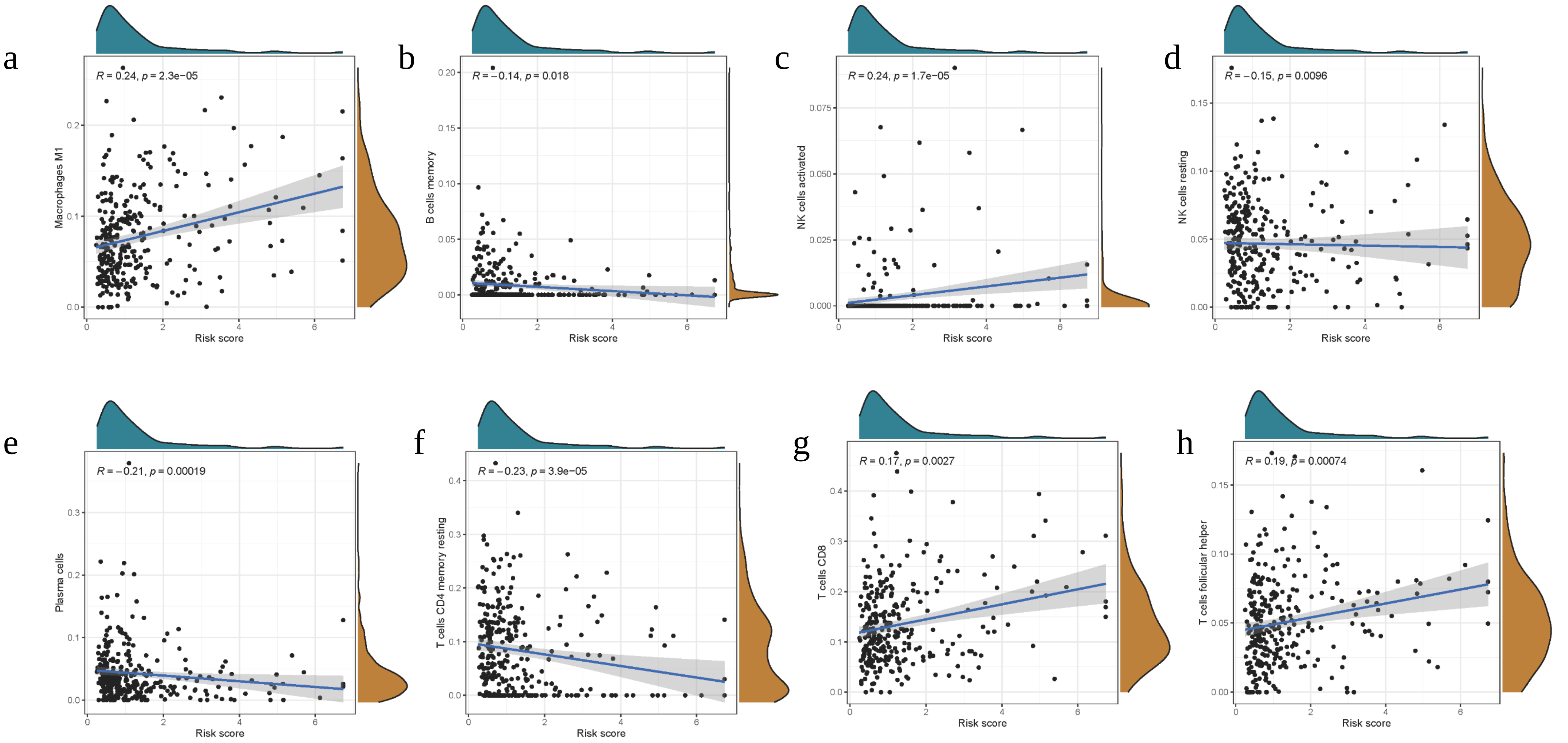

Figure S5 Correlation of immune cells with risk scores

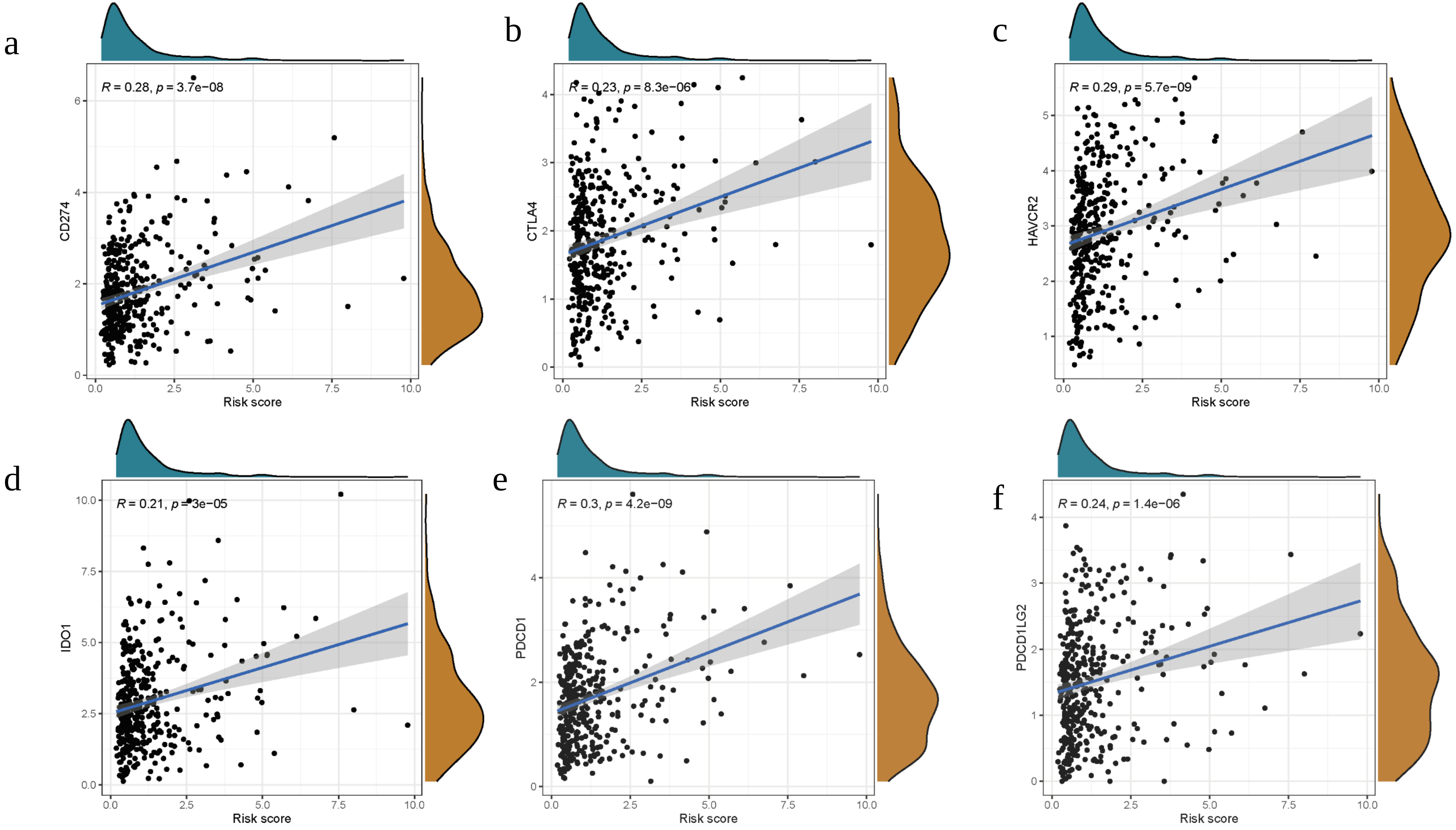

Figure S6 Correlation of immune checkpoints with risk scores
